# Reliable HPLC Determination of Aflatoxin M1 in Eggs

**DOI:** 10.1155/2013/817091

**Published:** 2013-08-01

**Authors:** Mostafa M. H. Khalil, Ahmed M. Gomaa, Ahmed Salem Sebaei

**Affiliations:** ^1^Chemistry Department, Faculty of Science, Ain Shams University, Cairo 11566, Egypt; ^2^QCAP Laboratory, Agriculture Research Center, Ministry of Agriculture, Giza 12311, Egypt

## Abstract

Aflatoxin M1 is the foremost metabolite of aflatoxin B1 in humans and animals, which may be present in animal products from animals fed with aflatoxin B1 contaminated feed. In this study a high performance liquid chromatography method for determination of aflatoxin M1 in eggs was described. The egg samples were diluted with warmed water and the toxin was immunoextracted followed by fluorescence detection. The average recovery of aflatoxin M1 at the three different levels 0.05, 0.1, and 0.5 **μ**g/kg varied between 87% and 98%. The method is linear from the limit of quantification 0.05 **μ**g/kg up to 3 **μ**g/kg levels. This method is intended for aflatoxin M1 analyses in eggs simply with minimum toxin lose, excellent recovery, and accurate results with the limit of detection 0.01 **μ**g/kg.

## 1. Introduction

**Figure 1:**
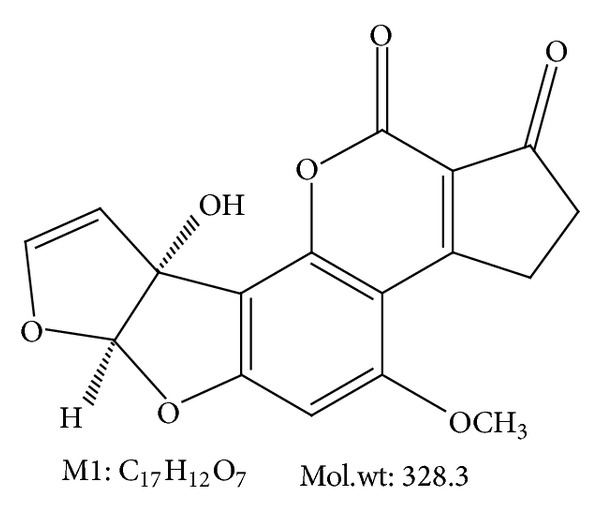
Structural formula for aflatoxin M1.

Mycotoxins are toxins produced by molds that cause diseases called mycotoxicosis [1]. Aflatoxins, the most common mycotoxins, are toxic metabolites produced by certain fungi that can occur in foods and feeds. Aflatoxin M1 (AFM1) is usually considered to be a detoxication byproduct of aflatoxin B1 and it is also the hydroxylated metabolite present in animal products that eat foods containing the aflatoxin B1 toxin. Aflatoxin M1 is cytotoxic and its acute toxicity is similar to that of aflatoxin B1 [2] and was classified in Group 2B as possibly carcinogenic to humans [3]. AFM1 is very slightly soluble in water, freely soluble in moderately polar organic solvents and insoluble in non-polar solvents. AFM1 is unstable to ultraviolet light in the presence of oxygen, pH (<3, >10) and oxidizing agents. On the other hand AFM1 has an intensely fluorescent in ultraviolet light [4]; its structural formula is given in Figure 1. Processing and storage cause a little effect on AFM1 content in milk and milk products [2, 5, 6].

AFM1 has been found worldwide in a range of animal products, including milk and milk products types, eggs, meat, and meat products [7–14].

Several chromatographic methods for AFM1 determination in various commodities include ELISA and flow-injection immunoassay system [15–17], HPLC methods with solid phase and immunoaffinity separations [18, 19], and other chromatographic post column, mass, and tandem mass spectrometry detection [20–22]. This study develops an HPLC method using immunoaffinity column with rapid and reasonable high test recovery comparable with other intended methods of analyses. 

## 2. Materials and Methods

### 2.1. Chemicals and Materials

All chemicals and reagents were of HPLC and analytical grade. Deionized water that was used throughout the experiments was generated by Milli-Q A10 FOCN53824k. Methanol and acetonitrile were (Lab-scan) (HPLC), with assay >99%. Standard of aflatoxin M1 (>98%) solution and Visiprep SPE vacuum manifold were purchased from Sigma Aldrich. Afla M1 immunoaffinity column was purchased from VICAM for HPLC aflatoxin M1 analysis (G1007). All performance parameters and statistical experiments were applied on chicken eggs samples.

### 2.2. Standard and Calibration Preparation

1 mL of 10 *µ*g/mL of AFM1 in acetonitrile was diluted in 10 mL volumetric flask with acetonitrile to obtain 1 *µ*g/mL as a working solution. Stock and working solution were kept in freezer at −20°C, the expiry date of the standard as indicated in the certificate of analysis. 0.05, 0.1, 0.5, 1.0 and 10 *µ*g/L are prepared by diluting the working standard in acetonitrile : water (30 : 70 v/v), 100 *µ*L of the solution was subjected to HPLC analysis and the correlation coefficient must be greater than 0.99.

### 2.3. Sample Preparation

5 grams of well-homogenized egg was added into 250 mL plastic bottle and 80 mL water added to the plastic bottle and the mixture warmed before analysis to 45°C for 30 min and centrifugated at 4000 rpm for 10 min. After filtration of the aliquot over small piece of cotton to separate fat (upper) layer from defatted phase, defatted solution passed completely through Afla M1 affinity column at a rate of about 1-2 drops/second until air comes through column. It should take 20 min for egg to flow through the column. The flow slowed down using the stopcock. Column was removed from plastic syringe barrel then 10 mL of purified water added through the column for washing with a rate of about 1-2 drops/second. Affinity column was eluted by passing 1.5 mL acetonitrile: methanol (60 : 40 v/v) through column at a rate of about 2-3 drops/second and collecting all of the sample elution (1.5 mL) in glass cuvette then 0.5 mL purified water through column at the same rate and collecting all of the sample eluation (1.5 + 0.5 mL) in the same glass cuvette (2 mL total volume). Vortex used to homogenize the eluation and 100 *µ*L of it were injected onto HPLC.

### 2.4. HPLC Analysis

High performance liquid chromatography instrument model HP Agilent 1200 series from Germany equipped with quaternary pump (G1311A), vacuum degasser (G1379B), autosampler (G1313A), and fluorescence detector Agilent 1260 infinity/1200 series (G1321A), analytical column: Agilent Eclipse plus C18 5 *µ*m 4.6 × 250 mm. Software: Chemistation for LC, Rev. B. 04.03 [16]. HPLC-pump flow rate: 0.8 mL min^−1^. AFM1 mobile phase : acetonitrile 30 : water 60 : methanol 10 (v/v/v). Detector parameters: fluorescence detector at (360 nm excitation, 440 nm emission).

### 2.5. Statistical Analysis

The method described was developed and optimized for all procedure steps with statistical treatments that enhance and optimize the test recovery, minimize time and reagents, and reduce matrix interferences as possible. Firstly AFM1 statistically optimized to be extracted in one rapid and efficient step. Regarding that eggs considered to be one of the most important food and very heavy and difficult matrix for analytical chemistry separation techniques, we secondly minimize the matrix as possible using the most prober immunoaffinity separation criteria. Finally AFM1 peak was chromatographically separated well.

## 3. Results and Discussion

### 3.1. Optimization of the HPLC Determination 

#### 3.1.1. Extraction

AFM1 contamination been reported in egg samples [23, 24], so a rapid and simple HPLC method was developed in this study for AFM1 determination in eggs. Relatively polar solvents are the most efficient solvents that have been used for extracting mycotoxins and water offers higher extraction efficiencies in mixtures, by increasing penetration of the organic solvent [25, 26]. Since AFM1 is very slightly soluble in water [2, 27], a suitable amount of water (80 mL) was added for extraction from egg tissues and it is the lowest amount facilitating the flow over the manifold system without blocking the immunoaffinity column taking into account the heavy density and viscosity of egg samples.

#### 3.1.2. Effect of Temperature and Time

**Figure 2:**
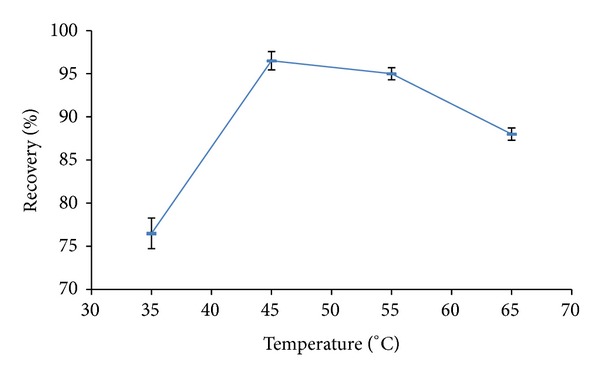
Effect of various extraction temperatures on AFM1 recovery (mean ± SD, *n* = 2).

**Figure 3:**
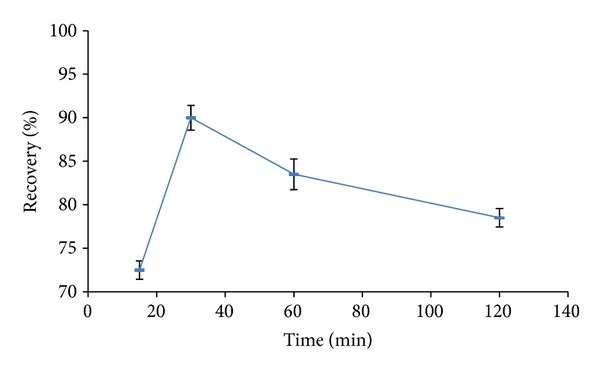
Effect of various times of incubation on AFM1 recovery (mean ± SD, *n* = 2).

In order to optimize the best extraction conditions the effect of temperature and time were checked for highest recovery yield which was expressed by mean recovery from two replicates for each experiment result.  Figure 2 exhibits that the extraction of AFM1 from eggs increased generally as the extraction temperature increased (where the time is fixed to 40 min. for the four results) till it reached the maximum at 45°C and the recovery decreased insignificantly till 65°C.  Figure 3 exhibits a significant recovery trend relative to the time of extraction (where the temperature fixed at 40°C). Recovery reached a maximum value at 30 min. and decreased significantly till the incubation time 120 min. This study suggested that an efficient extraction could be with the application of 45°C and 30 min. for best enhanced recovery.

#### 3.1.3. Elution

**Figure 4:**
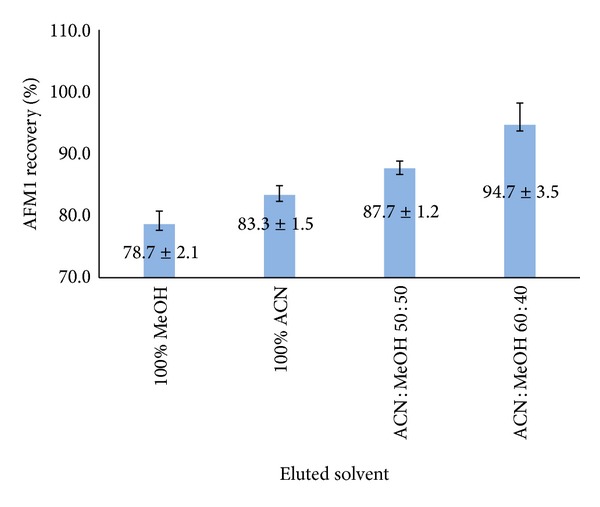
Selection of eluted solvent of methanol- (MeOH-) acetonitrile (ACN) for spiked egg samples (mean ± SD, *n* = 3, and SD expressed by error bars) at a level of 0.05 *μ*g/kg.

Various amounts from relatively polar solvents (acetonitrile and methanol) were tested and tried out for highest AFM1 test recovery in elution step and the recovery expressed as average recovery from 3 replicates for each solvent (Figure 4). All solvents offer test recovery in agreement with the European Union Commission Directive no. 401/2006 [28] from 70% to 110% for the range above 0.05 *μ*g/kg but the best recoveries were obtained from the mixture acetonitrile : methanol (60 : 40 v/v) as reported.

#### 3.1.4. HPLC Analysis

**Figure 5:**
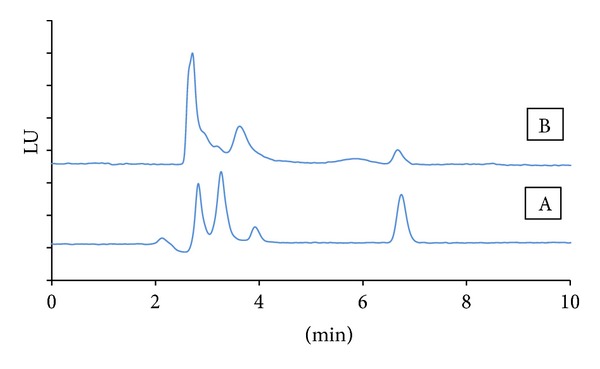
AFM1 HPLC-FLD chromatograms for 0.5 *μ*g/kg standard (A) and 0.05 *μ*g/kg egg spiked sample (B).

AFM1 was separated sufficiently without matrix interferences with the optimum mobile phase acetonitrile : water : methanol (30 : 60 : 10 v/v/v) which delivers fast AFM1 peak release where the method takes only 10 minutes and good symmetry (S > 0.85) for peak shape (Figure 5) with Agilent Eclipse plus C18. Chromatographic separation was performed with 0.8 mL/min so the method used minimal reagent quantities. The method shows excellent linearity with correlation coefficient 0.99995 with minimum variation in the calibration curve obtained from five levels 0.05, 0.1, 0.5, 1.0, and 10 *µ*g/L.

### 3.2. Method Validation (Fit for Purpose Approach)

Includes all of the procedures that demonstrate that a particular method used for quantitative measurement of analytes is reliable and reproducible for the intended use. EURACHEM [29, 30] and FDA [31] guidelines were followed for checking the method validation performance parameters which are summarized in Table 1.

#### 3.2.1. LOQ and LOD

Limit of quantification (LOQ) is the lowest amount of an analyte in a sample that can be quantitatively determined with suitable precision. The accuracy analyte peak (response) was identifiable, discrete, and reproducible with a precision of 9%. Limit of detection (LOD) is the minimum concentration of analyte that can be detected with acceptable certainty, though not quantifiable with acceptable precision and statistically determined as a trice of the standard deviation of sample blanks spiked at lowest acceptable concentration measured (Table 1). The method LOQ (0.05 *μ*g/kg) was represented at the lowest European Union MRL's [28] for AFM1 in milk and milk products and it is worth to mention that there is no regulations for AFM1 in eggs. The method was sensitive, with a detection limit 0.01 *μ*g/kg better than that reported by [32, 33]. 

#### 3.2.2. Precision and Trueness

Trueness is the degree of agreement of the mean value from a series of measurements with the true value or accepted reference value related to systematic error (bias). The method trueness was checked by certified reference material, proficiency test for milk powder matrix instead of egg by the same method because of unavailability and statistical trueness calculation which was estimated by spiked samples at different levels on eggs samples and bias expressed as absolute relative difference percent (RD%) must not exceed 20% (Table 1). Precision is degree of agreement of replicate measurements under specified conditions. The precision is described by statistical methods such as a standard deviation or confidence limit and less precision is reflected by a larger standard deviation and was classified as repeatability and reproducibility which was shown to be 9% and 13%, respectively, less than 20% in agreement with EU guideline 96/23/EC [34] and less than 15% in agreement with FDA guideline [31].

#### 3.2.3. Method Linearity and Test Recovery

Method linearity was checked by making recovery tests at three different levels of 0.05, 0.1, and 0.5 *μ*g/kg on eggs samples. Method was found to be linear from the limit of quantitation, 0.05, up to 0.5 *μ*g/kg with a strong correlation coefficient 0.99984. The check for method linearity performed with test recoveries for six replicates at the three different levels on eggs samples. As reported in Table 1, the method has excellent recoveries which varied between 87% and 98% at levels of 0.1 and 0.5 *μ*g/kg, respectively, which is in agreement with [28] between 70% and 110% and better than that reported by [11, 35].

#### 3.2.4. Uncertainty Measurement

The parameter associated with the result of a measurement that characterizes the dispersion of the values that could reasonably be attributed to the measured value. Uncertainty was estimated (at 95% confidence level and coverage factor of *k* = 2) to be in the range of ±33. Bias reported from uncertainty using *t*-test statistical calculations shows that the method recovery is significantly different from 100%, so the analytical result must be reported correctly for recovery for controlling compliance according to EURACHEM Guide for Quantifying Uncertainty [36].

## 4. Conclusion

Mycotoxins have serious effects on humans and animals. Thus, for ensuring food safety and monitoring the AFM1 hazards in animal products like eggs, a quick and accurate method was presented. This study aimed to optimize HPLC method with less interference, lowest reagent quantities, being safer for technicians, and being more easy to use. Several method validation parameters including LOQ, LOD, linearity, precision, trueness, test recoveries, and uncertainty measurement were checked for method performance. Characteristics of performance parameters indicate that the method is capable of determination of AFM1 in eggs with excellent analytical results and is recommended for food safety monitoring programs.

## Figures and Tables

**Table 1:** Trueness calculations and recoveries (mean ± SD, *n* = 6) for 3 spiking levels in eggs, LOD, LOQ, CRM^1^, and PT^2^ for AFM1.

Mycotoxin	Commodity	Spiking level (*µ*g/kg)	Recovery (%)	*X* − T*	Bias (RD %)^#^	LOD (*µ*g/kg)	LOQ (*µ*g/kg)	FAPAS CRM T04120 Accepted range (0.33–0.84) *µ*g/kg	FAPAS PT T04124 Assign value (0.505 *µ*g/kg)
AFM1		0.05	95.3 ± 9.3	2.3	4.7				0.29, *Z*-score = −1.9
	Eggs	0.10	98.0 ± 6.0	2.0	2.0	0.012	0.05	0.64	
		0.50	87.1 ± 6.7	64.3	12.9				

^1^Certified reference materials.

^2^Proficiency test.

*X: expected value, *T*: mean value.

^#^Relative difference.
